# Investigating Surface Morphology and Subsurface Damage Evolution in Nanoscratching of Single-Crystal 4H-SiC

**DOI:** 10.3390/mi16080935

**Published:** 2025-08-14

**Authors:** Jianpu Xi, Xinxing Ban, Zhen Hui, Wenlan Ba, Lijuan Deng, Hui Qiu

**Affiliations:** 1 School of Intelligent Mechanical and Electrical Engineering, Zhongyuan University of Technology, Zhengzhou 450007, China; denglj417@zut.edu.cn; 2 School of Mechanical and Electrical Engineering, Henan University of Technology, Zhengzhou 450001, China; xiaoba819@163.com (W.B.); qiuhuihere1987@163.com (H.Q.); 3 State Key Laboratory for High Performance Tools, Zhengzhou Research Institute for Abrasives & Grinding Co., Ltd., Zhengzhou 450001, China; huizhen@zzsm.com

**Keywords:** scratching, silicon carbide, brittle to ductile transition, removal mechanism, subsurface damage

## Abstract

Single-crystal 4H silicon carbide (4H-SiC) is a key substrate material for third-generation semiconductor devices, where surface and subsurface integrity critically affect performance and reliability. This study systematically examined the evolution of surface morphology and subsurface damage (SSD) during nanoscratching of 4H-SiC under varying normal loads (0–100 mN) using a nanoindenter equipped with a diamond Berkovich tip. Scratch characteristics were assessed using scanning electron microscopy (SEM), while cross-sectional SSD was characterised via focused ion beam (FIB) slicing and transmission electron microscopy (TEM). The results revealed three distinct material removal regimes: ductile removal below 14.5 mN, a brittle-to-ductile transition between 14.5–59.3 mN, and brittle removal above 59.3 mN. Notably, substantial subsurface damage—including median cracks exceeding 4 μm and dislocation clusters—was observed even within the transition zone where the surface appeared smooth. A thin amorphous layer at the indenter-substrate interface suppressed immediate surface defects but promoted subsurface damage nucleation. Crack propagation followed slip lines or their intersections, demonstrating sensitivity to local stress states. These findings offer important insights into nanoscale damage mechanisms, which are essential for optimizing precision machining processes to minimise SSD in SiC substrates.

## 1. Introduction

The processing requirements for single-crystal silicon carbide (SiC) substrates used in third-generation semiconductor chips are extremely stringent [1]. These substrates require ultra-smooth surfaces (surface roughness Ra < 0.2 nm) and must be free from subsurface damage (SSD); otherwise, chip performance and lifespan are compromised [2]. Critically, defects like surface/subsurface cracks directly degrade SiC’s defining superior electrical properties—including high carrier mobility and breakdown voltage—along with operational reliability [3]. However, single-crystal SiC—second only to diamond in hardness—exhibits exceptional chemical stability and is classified as a typical hard-brittle, difficult-to-machine material [4,5,6]. As a result, low polishing efficiency and the tendency to develop surface or subsurface defects severely hinder the advancement of wide-bandgap semiconductors [7,8,9]. Crucially, the applied normal load during machining critically governs the transition between ductile and brittle material removal regimes, directly influencing SSD generation [10]. A systematic investigation of the load-dependent material response is therefore essential for optimizing processing parameters to suppress defects [11]. To better understand defect formation and crack propagation mechanisms, in-depth research into the brittle–plastic material removal process of single-crystal SiC is urgently needed.

Research on defect and crack formation in single-crystal SiC machining primarily combines molecular dynamics (MD) simulation with scratch experiments [12,13]. A critical scratch depth governs material removal: Zhang et al. [14,15] confirmed ductile-mode removal for 6H-SiC at shallow depths, while Liang et al. [16] reported dislocation activation beyond a depth of 1.6 nm. Hu et al. [17] further found that scratching below 10 nm on 4H/6H-SiC mainly causes amorphous phase transformation with negligible dislocation or cracking. Regarding crack propagation, Gao et al. [18] showed via MD that cracks initiate from slip band extension following initial amorphization or dislocation damage and are stress-driven, dominated by maximum principal stress. Wang [19] and Tian [20] observed variations in scratch damage and dislocation behaviour across different atomic layers. The damage is strongly influenced by anisotropy [21]. Shi et al. [22] systematically clarified the removal mechanisms across crystallographic planes and orientations, showing periodicity at 30° intervals. Both Wu et al. [23] and Ni et al. [24] indicated that scratching the (0001) plane along the <1-100> direction produces the least SSD and best surface roughness. External conditions, such as environmental medium and temperature, also significantly affect damage and crack behaviour [25,26]. Wu et al. [27] found that higher temperatures shift 6H-SiC deformation from cleavage to plastic flow (through amorphisation and dislocations) but cause deterioration of the surface quality. Zhou et al. [28] clarified that a water film suppresses dislocation formation by breaking Si–Si bonds, thereby reducing friction and enhancing heat dissipation. Ban et al. [29] noted that oxidation reduces the surface hardness of 4H-SiC, effectively inhibiting crack formation and propagation. Wu et al. [30] further confirmed that tribochemical reactions enhance the material removal rate in 4H-SiC. In summary, although substantial progress has been made in understanding crack propagation in single-crystal SiC, the mechanisms governing SSD formation under varying loads—especially the thresholds for defect generation and plasticity-dominated removal—remain insufficiently explored.

Accurate characterisation of crack propagation requires effective detection of crack-like defects, with the choice of detection method being critical. Research shows that subsurface crack-like defects can form even when the surface appears intact [31]. Various methods have been employed to characterise SSD in single-crystal SiC: photochemical etching combined with molten alkali etching and ultraviolet photoluminescence reveals SSD as black defects [32]; X-ray diffraction (XRD) and dislocation density analysis are used to examine defects [33]; Mueller matrix spectroscopic ellipsometry quantifies the SSD layer thickness in 6H-SiC [34]; and Raman spectroscopy has been applied to analyse scratch characteristics and the material removal mechanism in 4H-SiC [35,36]. Tan et al. [37] further integrated Raman spectroscopy with transmission electron microscopy (TEM), revealing subsurface damage mechanisms in SiC at the atomic scale. Scanning electron microscopy (SEM) and TEM analyses demonstrate that chemical mechanical polishing (CMP) introduces high-density lattice defects into the subsurface region, including nano-scratches, basal plane dislocation loops, Shockley-type stacking faults, and Y-shaped defects [38,39]. Li et al. [40] conducted multi-dimensional characterisation by combining SEM, atomic force microscopy (AFM), Raman spectroscopy, and TEM, identifying the critical depth of cut enabling ductile-mode material removal and clarifying the formation mechanisms of stacking faults and twins. Overall, analysing post-scratch surface morphology using SEM, together with in-depth subsurface structural characterisation via TEM, constitutes an effective approach for investigating SSD in single-crystal SiC [41].

This study investigated single-crystal 4H-SiC using variable-load nanoscratch experiments conducted with a nanoindentation system. The surface morphology of the scratches was analysed using SEM. Cross-sectional cuts of the scratches were then prepared through focused ion beam (FIB) milling, and SSD within the substrate after scratching was examined by TEM. Through comprehensive analysis, the mechanisms of damage formation and crack evolution in single-crystal 4H-SiC during diamond scratching were elucidated. This work provides detailed insight into the material removal mechanism, offering a theoretical foundation for the ultra-precision machining of wide-bandgap semiconductor substrates.

## 2. Materials and Methods

Single-crystal 4H-SiC, one of more than 200 known silicon carbide polytypes, possesses a hexagonal crystal structure [42,43,44]. Due to its significant advantages in crystal growth and defect control over other polytypes, and its capacity to serve as a substrate enabling excellent homoepitaxial and heteroepitaxial quality, 4H-SiC has become a widely used substrate material in the field of wide-bandgap semiconductors [45,46]. The 4H-SiC substrate used in this study was a 2-inch, N-type polished wafer (thickness: 0.35 mm), purchased from SICC Co., Ltd. (Jinan, China). It featured an ultra-smooth surface (surface roughness Ra = 0.2 nm) and was defect-free, ensuring that the influence of surface quality could be neglected during the scratching process.

**Figure 1:**
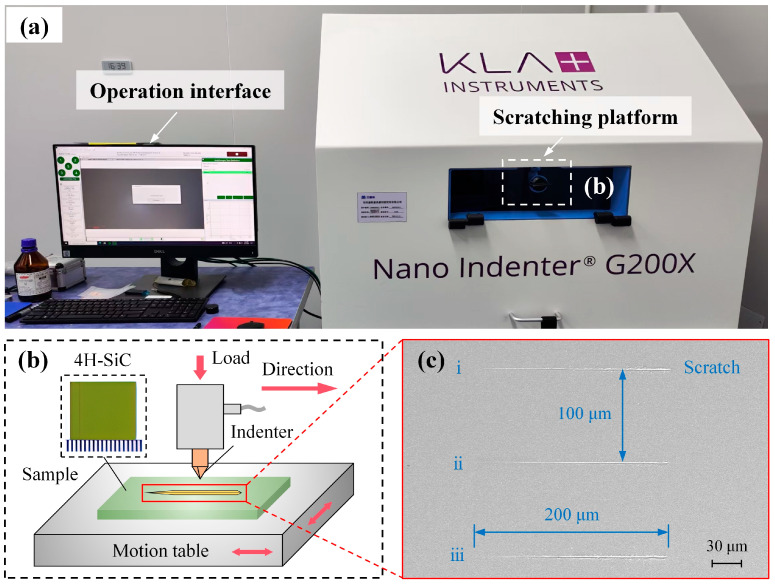
(**a**) G200X nanoscratch system, (**b**) schematic of the scratching principle, and (**c**) surface morphology after scratching.

Scratch testing was conducted using a KLA Instruments G200X nanoindenter (KLA Corporation, Milpitas, CA, USA), which has a maximum load of 500 mN and a displacement resolution of 0.01 nm (Figure 1a). The experimental parameters were as follows: a diamond Berkovich indenter (KLA Corporation, Milpitas, CA, USA) with a tip radius of 20 nm was used in an edge-forward orientation [31]. Tests were carried out on the Si-face (0001) of 4H-SiC, with the scratching direction aligned along the [11,12,13,14,15,16,17,18,19,20] crystal orientation [29] (Figure 1b). A ramp loading mode was applied, increasing the load linearly from 0 to 100 mN at a constant scratching speed of 10 μm/s over a scratch length of 200 μm. To minimise experimental error, three replicate scratches (labelled i, ii, and iii) were generated sequentially under identical testing conditions on the sample surface. The resulting scratch morphologies are shown in Figure 1c.

The scratch force, depth, and surface morphology of the three parallel scratches were compared, demonstrating good repeatability. Accordingly, the central scratch (Figure 1c(ii)) was selected for detailed analyses. Initially, the scratch morphology, microstructure, and surface defects were characterised using a ZEISS Gemini 300 scanning electron microscope SEM (Carl Zeiss AG, Oberkochen, Germany). A FIB cross-section was first prepared at a distance of 45 μm from the scratch initiation point and designated as Cross-section I. Two additional cross-sections were then prepared sequentially at 25 μm intervals and designated as Cross-section II and Cross-section III, respectively. This sectioning strategy was designed to allow observation of the subsurface damage evolution in both the ductile removal regime and the brittle-to-ductile transition zone. Finally, the subsurface damage characteristics of the three cross-sections were examined using transmission electron microscopy (TEM).

## 3. Results

### 3.1. Scratching Characteristics

**Figure 2:**
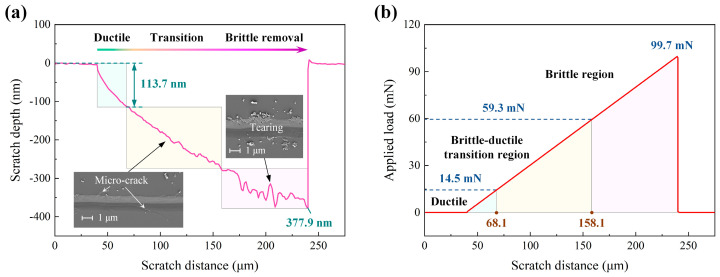
Curves of scratch depth (**a**) and applied load (**b**) versus scratch distance (note: the scratch depth values in (**a**) are negative according to the instrument’s coordinate system, while the text descriptions report the depths as positive magnitudes).

Figure 2 illustrates the scratching behaviour of single-crystal 4H-SiC under a diamond Berkovich indenter. As shown in Figure 2a, the scratch depth increases with the applied force, although the trend varies distinctly across depth intervals. When the scratch depth is less than 113.7 nm, the depth–distance curve remains smooth and free from significant fluctuations. According to the findings of Ban et al. [29], material removal in this stage is predominantly ductile, as evidenced by a smooth scratch surface with no visible defects; this region is therefore defined as the ductile removal regime. Figure 2b shows that the corresponding scratch length in this regime is 28.1 μm (following a 40 μm non-contact region) under an applied load of 14.5 mN. When the scratch depth exceeds 113.7 nm but remains below 278.1 nm, the depth–distance curve begins to exhibit fluctuations, which intensify as the depth increases. This stage represents the brittle-to-ductile transition, characterised by a surface that changes from smooth to one with minor defects such as micro-cracks and micro-tearing [47]. Beyond the 278.1 nm depth (corresponding to a load of 59.3 mN), the depth–distance curve shows pronounced oscillations, and the surface displays prominent defects, including cracks and tearing, consistent with brittle material removal. This final stage is defined as the brittle removal regime and is known to negatively affect machining quality. In summary, material removal is primarily ductile below a load of 14.5 mN, primarily brittle above 59.3 mN, and occurs within a brittle-to-ductile transition regime between these two load thresholds.

### 3.2. Surface Morphology Analysis

**Figure 3:**
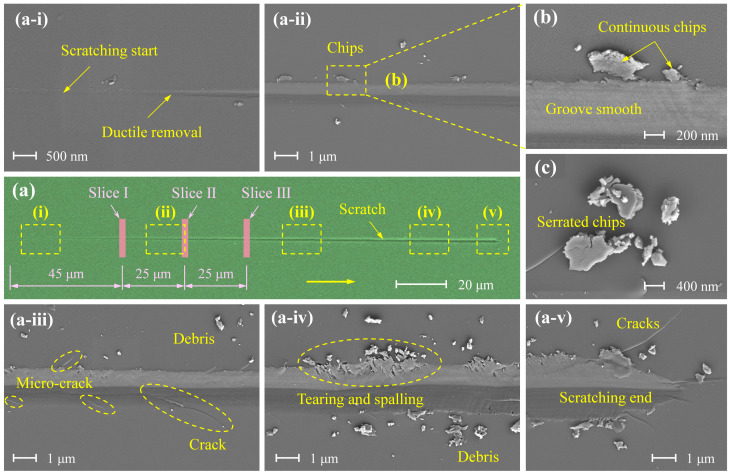
Scratch morphology under 100 mN varying load. (**a**) Overall scratch morphology. (**a-i**–**a-v**) Detailed views of scratch morphology at different scratching distances, respectively. (**b**,**c**) Magnified views of debris regions.

Figure 3 presents the morphological evolution of scratches on a 4H-SiC substrate caused by a diamond indenter under a linearly increasing load from 0 to 100 mN. It clearly illustrates the entire process—from initial contact to subsurface penetration—highlighting the surface material removal characteristics under varying load conditions. As shown in Figure 3a, the scratch progresses from shallow to deep and narrow to wide, indicating a full transition from ductile to brittle material removal, consistent with the analysis in Section 3.1.

Specifically, Figure 3(a-i) corresponds to the ductile removal regime, characterised by a shallow scratch depth, fine debris (material detached from the single-crystal SiC substrate) along the scratch edges, and a smooth, defect-free surface. Figure 3(a-ii,a-iii) represent the brittle-to-ductile transition regime, where both the scratch depth and width increase, the debris changes into blocky chips, and micro-cracks begin to initiate and propagate along the scratch surface. These defects become more pronounced with increasing depth, eventually resulting in macroscopically visible cracks, as shown in Figure 3(a-iii). Figure 3(a-iv,a-v) correspond to the brittle removal regime, exhibiting a substantial increase in both scratch width and depth. In addition to cracks, tearing, and spalling are observed along the scratch edges, accompanied by severe surface defects and damage. This morphological analysis supports the trends observed in the scratch depth–distance curve. During the ductile removal stage, fine chips form with no notable defects, indicating a continuous and stable material removal process. In the brittle-to-ductile transition regime, the chips become blocky and micro-cracks begin to form, and then the material removal becomes intermittent, resulting in fluctuations in the depth–distance curve. In the brittle removal regime, material is primarily removed through bulk spalling, producing large-scale defects and severe surface damage. The removal process in this stage is highly intermittent, leading to significantly reduced surface quality.

Figure 3b,c show the debris morphology characteristics at different material removal stages. Figure 3b corresponds to the ductile removal stage (or shallow scratching stage), where debris is distributed along both sides of the scratch groove. Some particles remain attached to the substrate, and the groove surface appears relatively smooth. In contrast, Figure 3c illustrates features typical of the brittle removal stage, with debris detached as large chunks exhibiting irregular edges. This morphology indicates an unstable removal process and a markedly increased surface roughness of the substrate. Therefore, observation of the debris morphology provides a reliable basis for identifying the dominant material removal mode.

### 3.3. Subsurface Damage Analysis

#### 3.3.1. FIB Sampling

**Figure 4:**
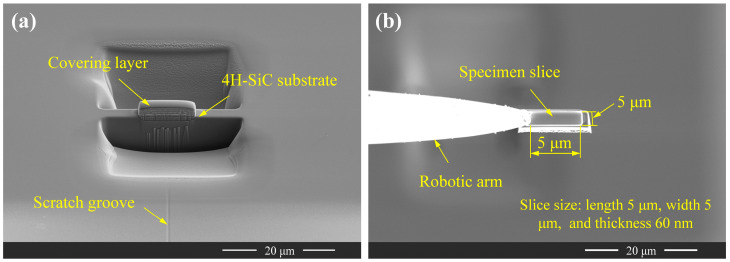
FIB sample preparation (**a**) and lift-out process (**b**) for 4H-SiC.

Following SEM characterisation of the scratch, FIB lift-out was performed at three designated locations on the scratch—labelled Slice I, Slice II, and Slice III—as shown in Figure 3a. The detailed procedure is illustrated in Figure 4. The sampling sites were aligned perpendicular to the scratching direction (Figure 4a), and the resulting TEM lamellae measured 5 μm × 5 μm × 60 nm (length × width × thickness) (Figure 4b).

**Figure 5:**
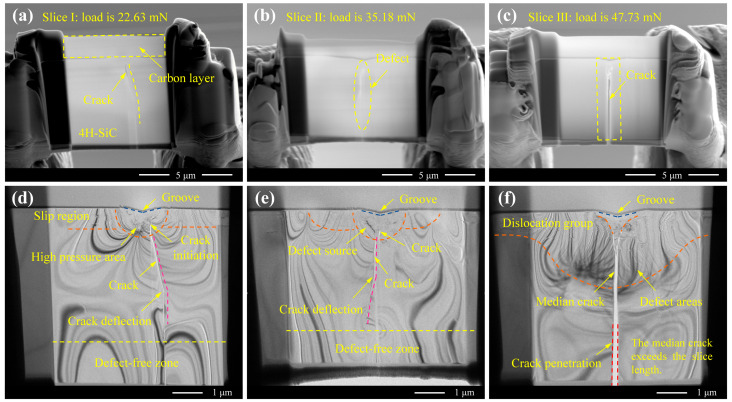
TEM images at different scratching positions. (**a**–**c**) Sections at 45 μm, 70 μm, and 95 μm from the scratch initiation point, respectively. (**d**–**f**) Magnified views of (**a**–**c**), respectively.

Figure 5 presents FIB cross-sections and corresponding TEM images at various scratch distances. A comparison of Figure 5a–c reveals distinct crack propagation directions and lengths at 45 μm, 70 μm, and 95 μm from the scratch initiation point. These positions correspond to applied loads of 22.63 mN, 35.18 mN, and 47.73 mN, respectively, as derived from the relationship shown in Figure 2b. Figure 5d–f clearly show that the substrate subsurface is composed of three distinct regions: a defect-concentrated region near the surface, localised around the scratch; an undamaged region deeper within the substrate, where the crystal structure remains intact; and a transitional region connecting the two. Furthermore, Figure 5f shows that the median crack propagates across the entire lamella (length: 5 μm), indicating that the crack length exceeds 5 μm at this point. To observe the subsurface damage morphology in greater detail, high-magnification TEM imaging was performed on each of these three regions. The corresponding analysis is provided in Section 3.3.2.

#### 3.3.2. Subsurface Damage

1. 1. Cross-section I

**Figure 6:**
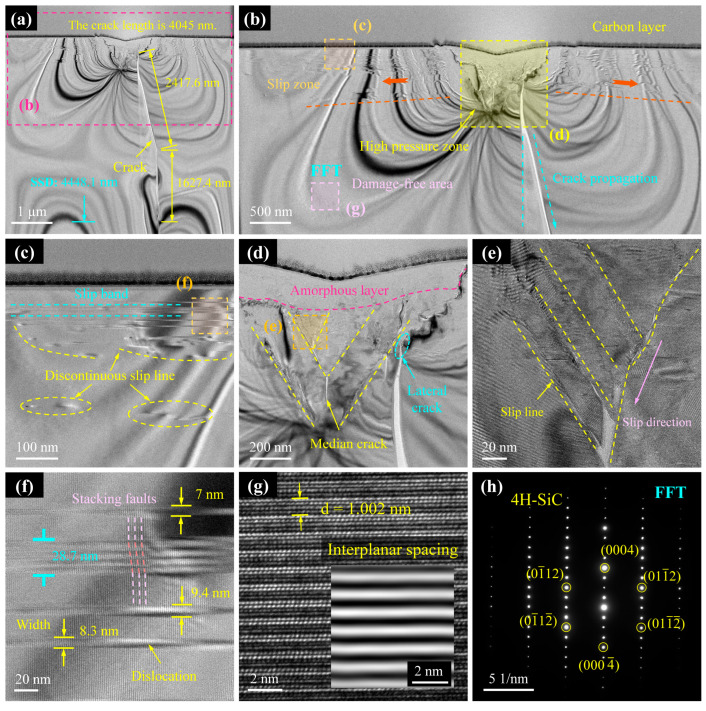
TEM results of subsurface damage in Cross-section I. (**a**) TEM images of subsurface after FIB slicing. (**b**) Enlarged images of (**a**). (**c**,**d**,**g**) Enlarged images of (**b**). (**e**) Enlarged images of (**d**). (**f**) Enlarged images of (**c**). (**h**) Diffraction spots obtained at location FFT.

Figure 6 presents the TEM analysis of Cross-section I, revealing detailed subsurface damage characteristics. As annotated in Figure 6a, the median crack has a total length of 4045 nm, while the subsurface damage layer extends to a depth of 4448 nm, measured from the crack propagation terminus. Notably, the crack does not propagate vertically downward from the scratch tip; instead, it initially extends 2418 nm laterally to the right before deflecting downward by a further 1627 nm. Analysis of the cross-section position indicates that this region lies within the brittle-to-ductile transition zone. Although the scratch surface appears smooth and intact, substantial subsurface damage remains—an observation consistent with the findings of Huang et al. [31]. This result confirms that surface morphology alone is insufficient for accurately assessing processing quality.

Figure 6b reveals a high-pressure zone beneath the scratch groove, which serves as the origin of subsurface defects and damage. From this zone, slip band clusters extend laterally, while cracks propagate in the longitudinal direction. Crystalline regions beyond the high-pressure zone retain an intact lattice structure (Figure 6g) and remain unaffected. The slip bands comprise both continuous and discontinuous slip lines, formed by lattice stacking faults. Under compression by the diamond indenter, lattice slip occurs on both sides of the groove, generating dislocation pile-ups (Figure 6c) that subsequently initiate damage. These slip bands range in width from 7 to 9.4 nm (Figure 6f), extending radially from the scratch centre and terminating in defect-free regions. The observed radial distribution of slip bands arises from the steep stress gradient emanating from the high-pressure zone beneath the indenter; slip activity diminishes with increasing radial distance as the resolved shear stress falls below the critical value required for dislocation motion. As shown in Figure 6d, an amorphous layer forms at the interface between the indenter and substrate. By inhibiting dislocation motion and crack propagation, this layer explains the contrasting observation of a smooth scratch surface despite the presence of significant subsurface damage. Beneath the amorphous layer lies the crack initiation and propagation zone, where crystals experience compressive lattice slip. Cracks nucleate at the intersections of slip lines [48], propagating either along the slip paths (Figure 6e) or extending downward as median cracks (Figure 6d). Figure 6g,h confirm that regions distant from the damage zone retain pristine crystal structures, with atomic arrangements consistent with the 4H-SiC lattice.

2. Cross-section II

**Figure 7:**
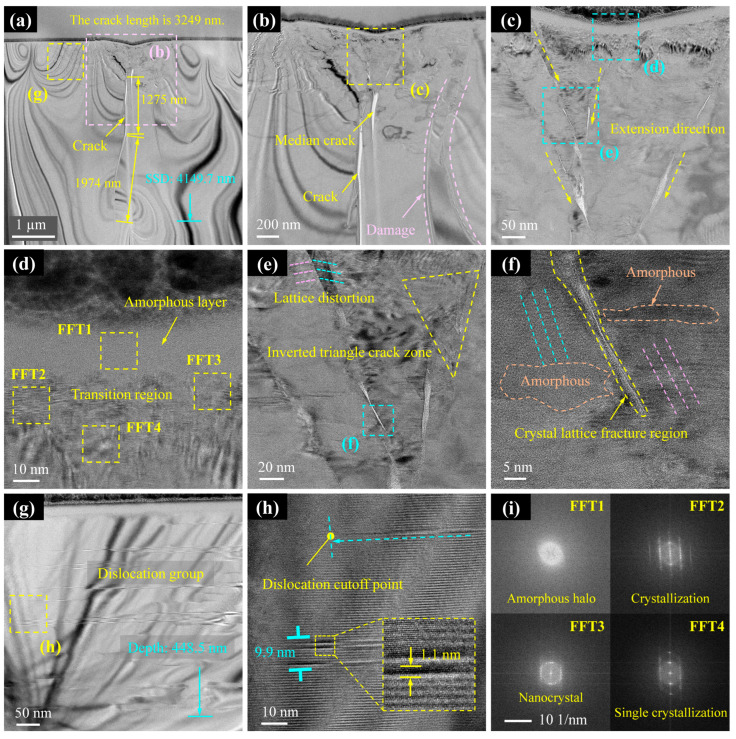
TEM results of subsurface damage in Cross-section II. (**a**) TEM images of the subsurface after FIB slicing. (**b**,**g**) Enlarged images of (**a**). (**c**) Enlarged images of (**b**). (**d**,**e**) Enlarged images of (**c**). (**f**) Enlarged images of (**e**). (**h**) Enlarged images of (**g**). (**i**) Diffraction spots obtained at locations FFT1, FFT2, FFT3 and FFT4.

Cross-section II experiences higher applied loads and scratch depths than Cross-section I, yet it displays reduced damage severity. As shown in Figure 7a, both the crack length (3249 nm) and the depth of the subsurface damage layer (4150 nm) are smaller than those observed in Cross-section I. Notably, the deflection direction of the median crack in Cross-section II is opposite to that in Cross-section I, curving leftward. This suggests non-deterministic crack propagation paths influenced by local stress states. Figure 7b,c confirms that the damage mechanisms remain consistent with those observed in Cross-section I: cracks propagate along slip lines or their intersections, while lattice slip persists near the median crack (Figure 7c), continuing to degrade the subsurface quality. Figure 7d reveals multiphase transformations beneath the scratch, where an amorphous surface layer transitions gradually into crystalline and nanocrystalline zones. Regions farther from the scratch retain a single-crystal phase. Slip line convergence zones form inverted triangular patterns (Figure 7e), with lattice distortion or fracture occurring at their intersections. Amorphisation and lattice rotation are observed along the flanks of slip lines (Figure 7f). In addition, dislocation clusters form along the scratch shoulders, extending to depths of approximately 449 nm (Figure 7g). Slip bands radiate outward from the scratch centre, progressively decreasing in both width and density until they disappear. Near their termination, individual slip bands measure approximately 1.1 nm in width (Figure 7h). In summary, Cross-section II exhibits similar damage morphology to Cross-section I; however, the depth of subsurface damage does not scale proportionally with the applied load.

3. Cross-section III

**Figure 8:**
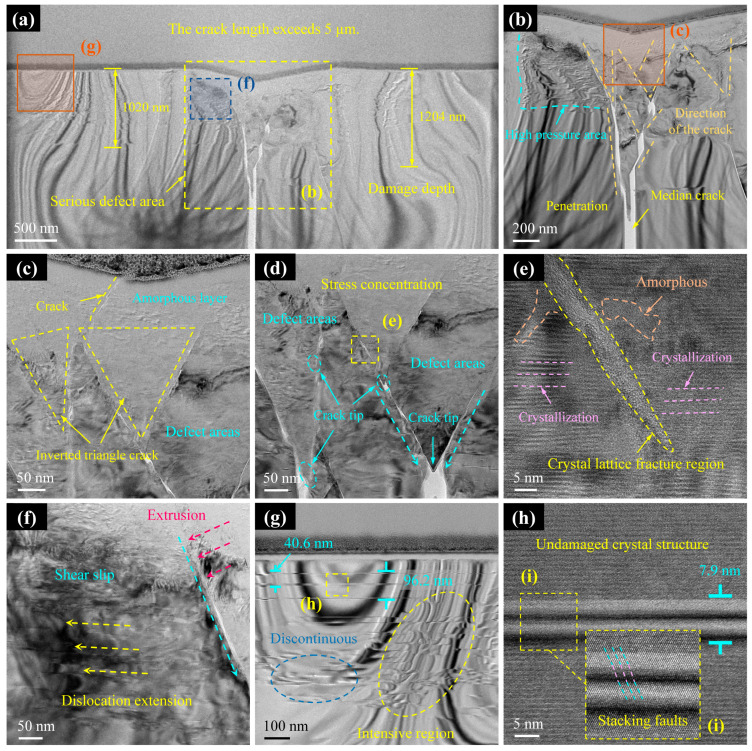
TEM results of subsurface damage in Cross-section III. (**a**) TEM images of the subsurface after FIB slicing. (**b**,**f**,**g**) Enlarged images of (**a**). (**c**) Enlarged images of (**b**). (**d**) Crack initiation zone. (**e**) Enlarged images of (**d**). (**h**) Enlarged images of (**g**). (**i**) Enlarged images of stacking faults.

As the applied load increased to 47.7 mN, the scratch depth also increased, and both surface and subsurface damage intensified significantly. The damaged morphology of Cross-section III is shown in Figure 8. As previously noted, the median crack in this section propagated entirely through the prepared specimen, exceeding the measurable range, and was confirmed to be longer than 5 μm. Compared to Cross-sections I and II, Cross-section III exhibited more severe subsurface impacts, with deeper damage zones and high-density dislocation clusters along the scratch flanks, extending to depths of 1204 nm (Figure 8a). Notably, the median crack in this section followed the longitudinal centreline of the scratch, a feature that distinguishes it from the previous cross-sections. As shown in Figure 8b, the direction and pattern of lattice slip induced by the diamond indenter pressing into the substrate remained consistent with those observed in Cross-sections I and II. Dense slip bands and associated defects were distributed along both sides of the scratch and extended downward, gradually diminishing in intensity with depth. The through-thickness crack caused structural fracturing of the specimen slice.

Figure 8c reveals an increased thickness of the amorphous layer beneath the scratch, where cracks originating from lattice slip propagated upward from within the substrate, penetrating the amorphous layer to reach the scratch surface. This behaviour is consistent with microcrack and crack formation during the brittle-to-ductile transition. Below the amorphous region, the defect zone became more degraded; however, the crack retained an inverted triangular distribution, with its tip representing the most likely site for larger crack nucleation to relieve shear stress (Figure 8d). The regions adjacent to the slip lines remained the primary zones of crystalline phase transformation, exhibiting both amorphisation and recrystallisation phenomena (Figure 8e). Figure 8f illustrates the mechanism of dislocation formation: shear stress generated by the diamond indenter caused lattice fracture and slip, with shear slip occurring on the left side of the slip line (aqua green arrow in Figure 8f), resulting in lattice misalignment and dislocation development [19,49]. Areas adjacent to the slip line concentrated in dislocation clusters, while more distant regions contained a mixture of continuous and discontinuous dislocations (Figure 8g). High-magnification observation of dislocations (Figure 8h) showed that stacking faults constituted their primary form. This analysis demonstrates that higher scratching loads promote the formation of more severe surface and subsurface defects, which are highly detrimental to machining quality control.

## 4. Discussion

This study offers comprehensive insights into the nanoscratching behaviour and subsurface damage mechanisms of single-crystal 4H-SiC. The identification of distinct material removal regimes—ductile, brittle-to-ductile transition, and brittle—based on scratch depth and applied load thresholds (<14.5 mN, 14.5–59.3 mN, >59.3 mN) aligns with established understanding of hard brittle materials. However, subsurface analysis revealed critical complexities that were not apparent from surface morphology alone.

The most significant finding is the presence of substantial subsurface damage, in-cluding deep median cracks (>4 μm) and dislocation clusters, even within the brittle-to-ductile transition zone where the scratch surface appears smooth and defect-free (e.g., Cross-section I) [50]. This confirms that surface smoothness is an inadequate indicator of processing quality, as severe subsurface degradation can remain undetected. The formation of a thin amorphous layer at the indenter–substrate interface plays a dual role: it inhibits immediate surface defect formation and dislocation motion—explaining the smooth surface appearance—but also acts as a site for stress concentration, promoting subsurface crack nucleation and propagation along slip lines or their intersections.

The subsurface damage mechanisms were consistent across the investigated cross-sections. Lattice slip under indenter compression generated dislocation pile-ups and slip bands radiating from the scratch centre. Cracks consistently initiated at slip line intersections or propagated along slip lines, forming median cracks that deflected non-uniformly (e.g., rightward versus leftward in Cross-sections I and II), indicating sensitivity to local stress states rather than deterministic crystal orientations alone. Phase transformations occurred directly beneath the scratch, transitioning from an amorphous surface layer to crystalline and nanocrystalline zones, with slip line convergence zones exhibiting lattice distortion, rotation, or fracture [24,51]. The depth of the subsurface damage layer (up to ~4.4 μm) and the extent of dislocation clusters (reaching ~1.2 μm) did not follow a simple linear relationship with increasing scratch load or depth, suggesting complex interactions within the stress field.

The observed damage evolution—including increasing crack length, dislocation cluster density and depth, and amorphous layer thickness with load—highlights the det-rimental effects of higher loads on both surface and subsurface integrity, which is espe-cially critical for precision semiconductor applications [29,52]. The brittle removal regime, characterised by extensive surface spalling, cracking, and tearing, is clearly unfavourable. Therefore, to achieve optimal machining quality control, material removal processes should operate within the purely ductile regime to minimise both visible and latent damage.

## 5. Conclusions

In this study, the formation mechanisms of surface and subsurface damage during material removal were investigated through variable-load nanoscratch experiments on single-crystal 4H-SiC. The following conclusions were drawn.

(1) Nanoscratching under a linearly increasing load revealed three distinct regimes: ductile removal (<14.5 mN), characterised by smooth surfaces and fine debris; the brittle-to-ductile transition, marked by increasing fluctuations in scratch depth, blocky chip formation, and micro-crack initiation; and brittle removal (>59.3 mN), exhibiting severe surface cracking, tearing, spalling, and significant depth oscillations.
(2) TEM analysis of FIB cross-sections demonstrated substantial SSD, including deep median cracks (>4 μm) and dislocation clusters (up to ~1.2 μm in depth), even within the brittle-to-ductile transition zone where the scratch surface appeared defect-free. This highlights the inadequacy of relying solely on surface morphology to assess processing quality.
(3) A thin amorphous layer formed at the indenter–substrate interface. While this sup-pressed surface damage and dislocation formation, it also concentrated stress, trig-gering subsurface cracks. These propagated along slip lines or their intersections (forming median cracks), with propagation direction sensitive to local stress. Beneath the amorphous layer, phase transitions occurred: amorphous, crystalline/nanocrystalline, and undamaged crystalline zones.
(4) With increasing scratch load or depth, the SSD layer and dislocation clusters did not exhibit a simple linear correlation, indicating complex stress field interactions. Nev-ertheless, the overall severity of damage—including crack length, dislocation density and depth, and amorphous layer thickness—showed a clear increasing trend with applied load.

Overall, these findings provide valuable insights into the mechanisms of material removal and the evolution of surface and subsurface damage during precision machining of single-crystal SiC, offering guidance for the advancement of wide-bandgap semicon-ductor substrate development.

## Data Availability

The original contributions presented in this study are included in the article. Further inquiries can be directed to the corresponding author.
